# Determinants of Effective Height in Morphologically Normal Tricuspid Aortic Valves: A Computed Tomography-Based Anatomical Study

**DOI:** 10.1093/icvts/ivag233

**Published:** 2026-08-20

**Authors:** Keiichi Ishida, Takashi Igarashi, Hitoshi Yokoyama, Kenichi Imasaka

**Affiliations:** Department of Cardiovascular Surgery, Fukushima Medical University, 1 Hikarigaoka, Fukushima, 960-1295, Japan; Department of Cardiovascular Surgery, Fukushima Medical University, 1 Hikarigaoka, Fukushima, 960-1295, Japan; Department of Cardiovascular Surgery, Fukushima Medical University, 1 Hikarigaoka, Fukushima, 960-1295, Japan; Department of Cardiovascular Surgery, Fukushima Medical University, 1 Hikarigaoka, Fukushima, 960-1295, Japan

**Keywords:** effective height, aortic valve complex, morphological parameter, electrocardiography-gated cardiac computed tomography, aortic valve repair

## Abstract

**Objectives:**

To analyse the relationship between effective height (eH) and other morphological parameters of the aortic valve complex when the aortic valve is closed.

**Methods:**

We retrospectively analysed 105 patients (mean age 70.7 ± 10.1 years) with morphologically normal aortic valves, roots, or ascending aortas who had undergone electrocardiography-gated cardiac computed tomography as a screening test for the coronary artery, from August 2014 to December 2018. Parameters of the aortic valve complex configuration (eH, geometric height (gH), aortic annular diameter (AN diameter), sinus of Valsalva diameter, sinotubular junction (ST-J) diameter, commissure height, and Valsalva height) in diastole were measured using Ziostation2 software. Pearson’s correlation was used to evaluate the associations between the eH and other parameters. Multiple regression analysis was performed to evaluate the variables independently associated with eH.

**Results:**

Significant associations were observed between the eH and gH, sinus of Valsalva diameter, ST-J diameter, commissure height, and Valsalva height (all *P* < .001) but not between eH and AN diameter (*P* = .227). Multivariable analysis showed that gH, AN diameter, and ST-J diameter were independently associated with eH (all *P* < .001; overall model fit: *R*^2^ = 0.739).

**Conclusions:**

Our findings demonstrate that eH is strongly associated with gH, which remained independently associated after adjustment, along with aortic annular and ST-J diameters. These results provide quantitative in vivo evidence regarding the anatomical determinants of eH in morphologically normal tricuspid aortic valves and may serve as anatomical reference data for future research on individualized approaches to aortic valve repair.

## Graphical Abstract

**Figure:**
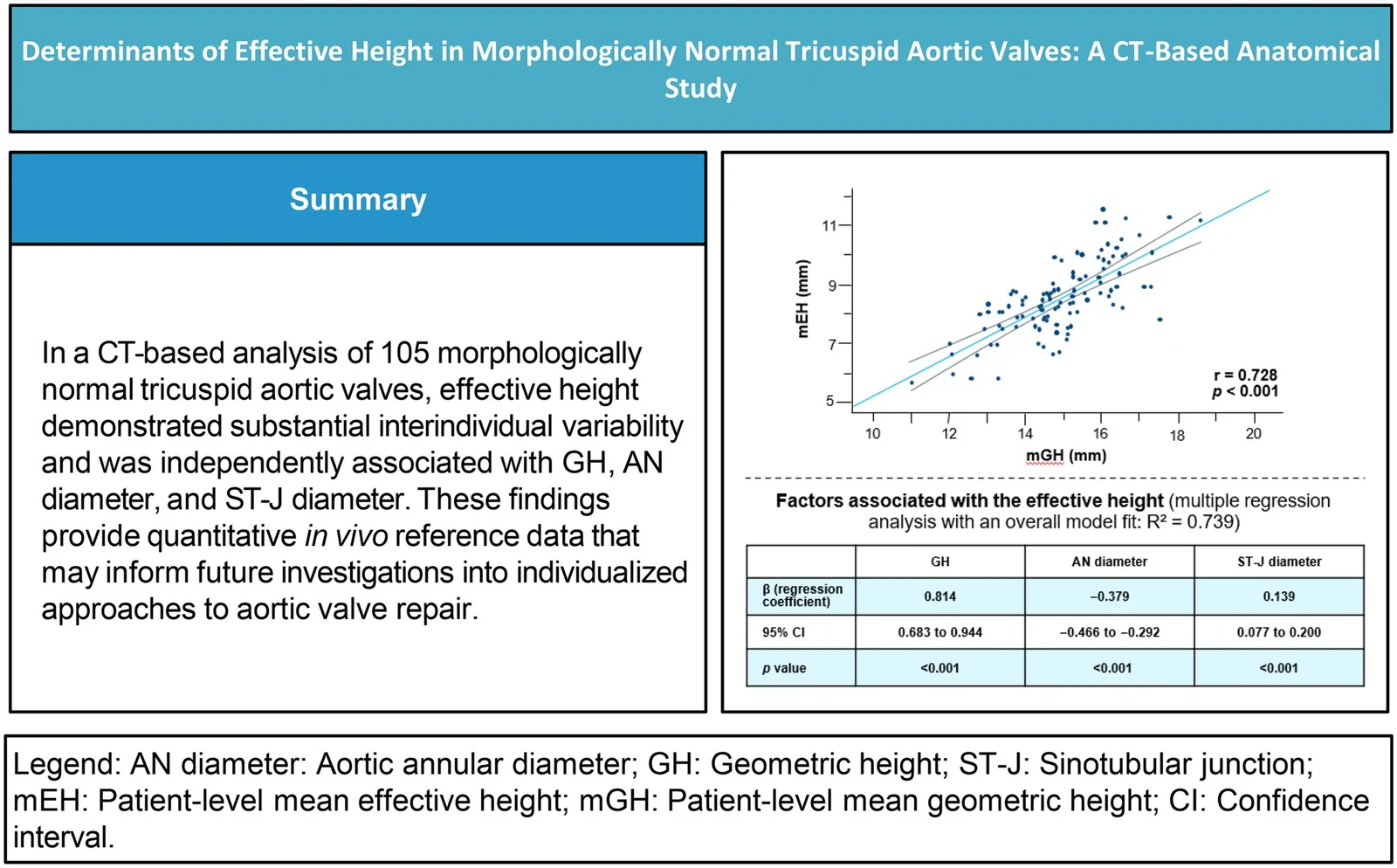

## INTRODUCTION

Recently, less invasive procedures such as transcatheter aortic valve replacement have expanded therapeutic options for patients with aortic valve disease. Nevertheless, surgical aortic valve replacement remains the current standard of care owing to its long-term outcomes. However, prosthetic valve implantation is inherently associated with several drawbacks, including thromboembolic events, bleeding complications related to lifelong anticoagulation, and prosthetic valve endocarditis.

To overcome these limitations, aortic valve repair (AVr), which preserves the native valve and restores its competence, is increasingly being performed, particularly in Western countries. Aortic valve repair is associated with favourable survival and low reoperation rates.^1^ It provides comparable or superior long-term results compared with aortic valve replacement. For instance, a significantly lower rate of valve-related adverse events is reported after AVr, with event-free survival of 82% at 10 years, versus that of 68% after aortic valve replacement.^2^ Despite these promising results, AVr tends to be mainly performed at experienced centres, and its indications, techniques, and standardization remain under discussion.

One major reason for the limited adoption of AVr is the complex 3-dimensional anatomy of the aortic valve complex, which makes repair technically demanding and difficult to standardize. Ideally, a successful AVr should aim to reproduce the morphology of a normal aortic root and cusp configuration to restore normal valve function. Accordingly, morphological analyses of the normal aortic valve complex may provide anatomical reference data for future refinement of AVr strategies.

The effective height (eH) of the aortic cusp is a key parameter for achieving a durable AVr.^3^^,^^4^ It provides a practical intraoperative target for cusp resuspension and deepens our understanding of the intricate 3-dimensional geometry of the aortic valve complex. Several studies have examined the anatomical factors influencing the eH.^4^^,^^5^ Nevertheless, individual variations in the relationship among the aortic root components suggest that the optimal eH may be patient-specific.

Conventionally, the eH is assessed by echocardiography or direct intraoperative measurements during cardioplegic arrest. However, few studies have investigated the relationship between the eH and morphological dimensions of the normal aortic valve complex using quantitative in vivo imaging. Recently, 3-dimensional electrocardiography (ECG)-gated cardiac computed tomography (CT) has been recognized as a highly reproducible modality for quantitative assessment of the valvular anatomy and has become the standard for valve sizing in transcatheter aortic valve replacement planning.

Therefore, we aimed to analyse the relationship between eH and other morphological parameters of the normal aortic valve complex using ECG-gated cardiac CT and to quantitatively characterize the anatomical determinants and variability of eH in morphologically normal tricuspid aortic valves.

## PATIENTS AND METHODS

### Patients

We retrospectively analysed a database of 154 consecutive patients who underwent ECG-gated cardiac CT as a preoperative screening test for the coronary artery during hospitalization in our department between August 2014 and December 2018. The exclusion criteria were as follows: bicuspid aortic valve, moderate or severe aortic regurgitation or stenosis, or aortic valve tumour; aneurysm of the aortic root or ascending aorta; history of cardiac surgery or replacement of the ascending aorta; and inappropriate image reconstruction. The final analysis included 105 patients with morphologically normal tricuspid aortic valves, and the configuration parameters of the aortic valve complex were measured. **Figure 1** presents the patient selection process.

**Figure 1.**
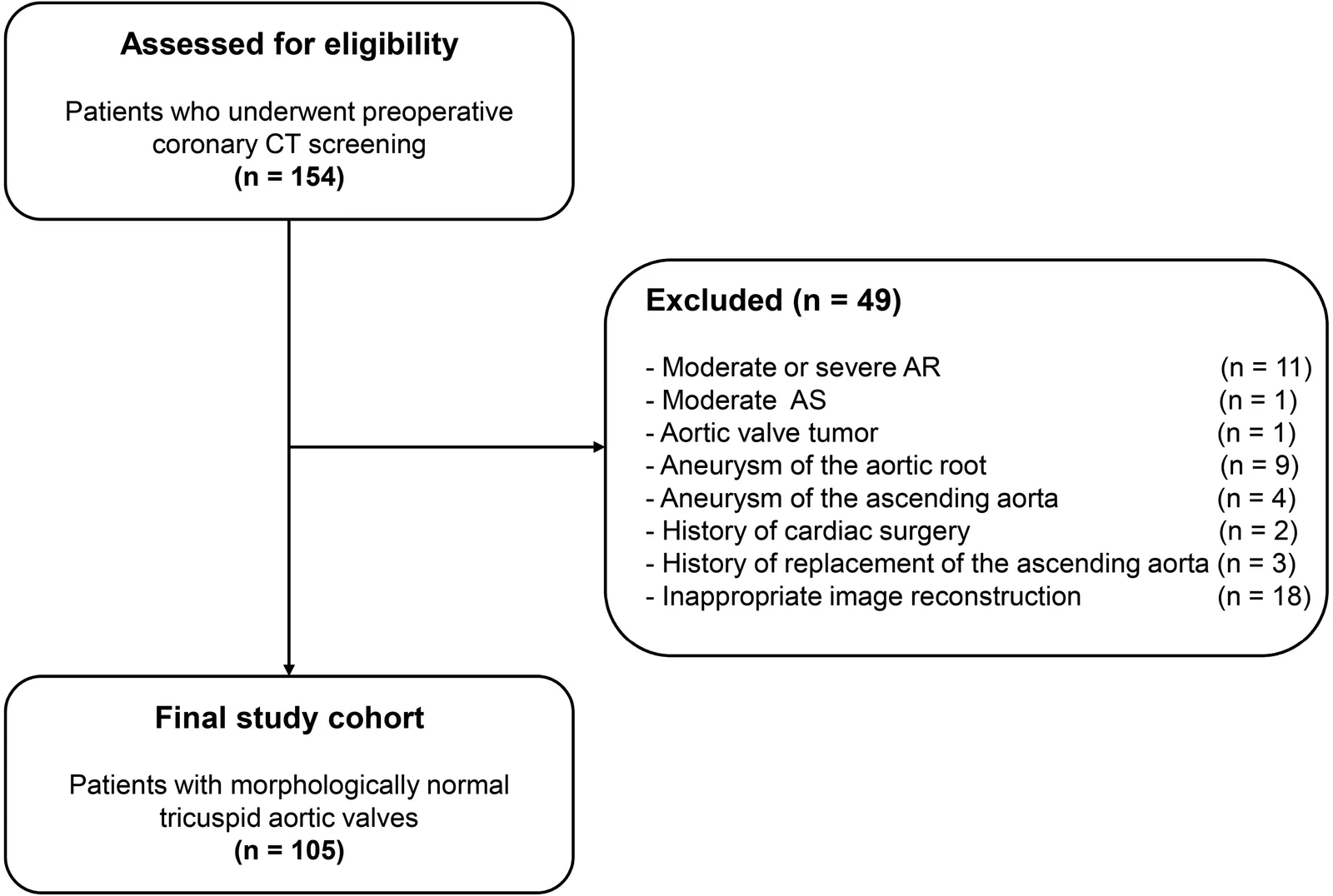
Patient Selection Flow Diagram. Among 154 consecutive patients who underwent ECG-gated cardiac computed tomography as a preoperative coronary screening examination, 49 were excluded according to the predefined exclusion criteria. The final cohort comprised 105 patients with morphologically normal tricuspid aortic valves. Abbreviation: CT, computed tomography

The study protocol was approved by the institutional review board (IRB) of Fukushima Medical University (IRB No. 30203, December 18, 2018), which waived the requirement for individual informed consent. Patients were informed at registration that their anonymized data may be used for research and were offered the opportunity to decline participation through an opt-out process publicized on the institutional website. This study was conducted in accordance with the ethical standards of the Declaration of Helsinki and the World Medical Association Declaration of Taipei. The storage, management, and use of research data were approved by the IRB and conducted under institutional oversight in accordance with the approved study protocol.

### CT measurements

Before December 2016, examinations were performed using a 64-slice multi-slice CT scanner (Aquilion 64 TSX-101A; Toshiba Medical Systems Co., Ohtawara, Japan), and after, they were performed on a 320 slice multidetector CT scanner (Aquilion ONE GENESIS; Canon Medical Systems Co., Ohtawara, Japan) using axial image acquisition with prospective ECG gating. The slice thickness of the reconstructed images was 0.5 mm. We analysed the CT data using Ziostation2 software (version 2.9.2.3; AMIN Co., Ltd., Tokyo, Japan; Ziosoft, Inc., Tokyo, Japan), which is regularly used for planning transcatheter aortic valve implantation or endovascular aortic repair at our institution. All measurements were performed using image data reconstructed at mid-diastole. Two independent observers specializing in cardiovascular surgery measured these parameters while blinded to each other’s measurements. We set the virtual basal ring plane connecting through the nadir of the 3 aortic valve cusps in the same manner as recommended for preprocedural CT imaging before transcatheter aortic valve implantation (**Figure 2**). The aortic annular (AN) diameter was measured as the area-derived diameter (=2×√(area/π)) of the basal ring plane. The sinus of Valsalva diameter was measured as the distance from each coronary sinus to the opposite interleaflet triangle on the sinus of Valsalva plane, the dimensions of which were the widest and parallel to the virtual basal ring plane. Next, we set 3 cross-sectional planes crossing through the coaptation point of the aortic valve cusps, each nadir of the cusp, and the opposite commissure vertical to the virtual basal ring plane. For each of the 3 planes, we measured the geometric height (gH) as the length of the curved line of the cusp, eH as the vertical distance from the virtual basal ring to the top of the cusp, commissure height as the vertical distance from the virtual basal ring to the top of the commissure, and Valsalva height as the vertical distance from the virtual basal ring to the inflection point located at the sinotubular junction (ST-J). We set the commissure plane crossing the 3 commissures and measured the commissure diameter as the area-derived diameter (=2×√(area/π)) of the plane. We also set the ST-J plane crossing the 3 inflection points positioned at the ST-J and measured the ST-J diameter as the area-derived diameter (=2×√(area/π)) of the plane.

**Figure 2.**
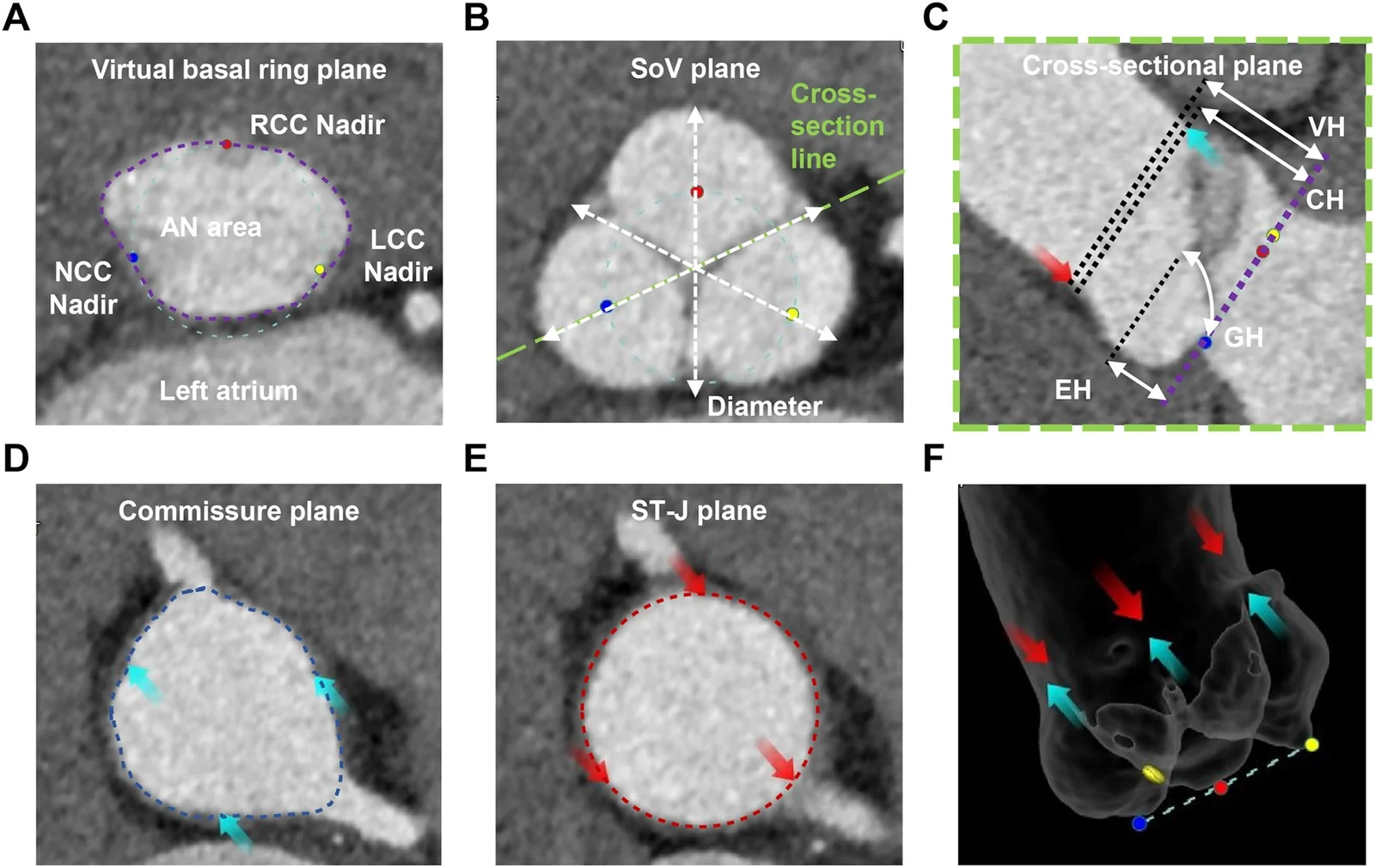
Computed Tomography Measurements of the Configuration of the Aortic Valve Complex. (A) An area measurement. The small red, yellow, and blue dots indicate the nadir of the RCC, LCC, and NCC, respectively. (B) SoV diameter measurement. (C) eH, CH, and VH. The tip of the red arrow indicates the inflection point located in the ST-J. The tip of the light blue arrow indicates the top of the commissure. (D) Measurement of the aortic area of the commissure plane. (E) Measurement of the aortic area of the ST-J plane. (F) Positional relationship of the virtual basal ring plane, ST-J, and commissure within the aortic valve complex. Abbreviations: AN area, aortic annular area; CH: commissure height; eH, effective height; gH, geometric height; LCC, left coronary cusp; NCC, non-coronary cusp; RCC, right coronary cusp; SoV, sinus of Valsalva; ST-J, sinotubular junction; VH, Valsalva height

### Statistical analysis

For patient-level analyses, cusp- or commissure-specific measurements were averaged for each patient to obtain representative mean values for eH, gH, sinus of Valsalva diameter, commissure height, and Valsalva height. Normally distributed continuous variables are presented as the mean ± standard deviation and categorical variables as number and frequency. The obtained data were tested for normality using the Shapiro-Wilk test. All statistical analyses were performed using the IBM SPSS 26 software (IBM Corp., Armonk, NY, United States). Analysis of variance was used to compare the differences in normally distributed variables, and the Friedman test was used for non-normally distributed variables. Pearson’s correlation coefficient was used to evaluate the association between eH and other parameters. In addition, multiple regression analysis was performed to identify the variables independently associated with eH. Statistical significance was set at *P* < .05. Candidate variables for multivariable regression included sex, body surface area, and major CT-derived parameters of the aortic valve complex. A stepwise variable selection procedure was used to construct the final model. Multicollinearity among predictors was assessed using correlation matrices and the variance inflation factor. Model performance was visually assessed using a scatterplot of observed versus predicted eH values. Intra-class correlation coefficients were used to assess the intra- and interobserver reliabilities for the mean eH, gH, aortic annular diameter (AN diameter), and ST-J diameter among the same randomly selected 30 patients.

## RESULTS

The mean age of the patients was 70.7 ± 10.1 years, and 81% were male (**Table 1**). The mean body height and body surface area were 163.5 ± 8.5 cm and 1.69 ± 0.19 m^2^, respectively. The mean phase of image reconstruction was 75%±4%.

**Table 1.** Baseline Characteristics

Variable	*n* = 105
Age (years)	70.7 ± 10.1
Male, *n* (%)	85 (81)
Body height (cm)	163.5 ± 8.5
Body weight (kg)	64.0 ± 12.4
Body mass index (kg/m^2^)	23.8 ± 3.7
Body surface area (m^2^)	1.69 ± 0.19
Hypertension, *n* (%)	83 (79)
Dyslipidaemia, *n* (%)	62 (59)
Diabetes mellitus, *n* (%)	25 (24)
Haemodialysis, *n* (%)	2 (2)
Chronic obstructive pulmonary disease, *n* (%)	28 (27)
Atrial fibrillation, *n* (%)	9 (9)
Cerebrovascular disease, *n* (%)	21 (20)
Coronary artery disease, *n* (%)	21 (20)
Heart disease, *n* (%)	2 (2)
Aortic aneurysm, *n* (%)	
Thoracic	22 (21)
Thoracoabdominal	1 (1)
Abdominal	56 (53)
Aortic dissection, *n* (%)	14 (13)
Peripheral arterial aneurysm, *n* (%)	12 (11)
Peripheral arterial disease, *n* (%)	13 (12)
Parameters of transthoracic echocardiography	
Ejection fraction (%)	63.2 ± 6.6
Left ventricular end-diastolic diameter (mm)	44.1 ± 5.6
Left ventricular end-systolic diameter (mm)	27.4 ± 5.0
Aortic regurgitation, *n* (%)	
None/trivial/mild	64/17/24 (61/16/23)

The dimensions of the aortic valve complex are listed in **Table 2**. The mean diameters for AN, sinus of Valsalva, commissure, and ST-J were 24.2 ± 1.9 mm, 34.8 ± 2.9 mm, 32.3 ± 2.9 mm, and 30.1 ± 2.7 mm, respectively. The mean ratios of the sinus of Valsalva, commissure, and ST-J diameters to the AN diameter were 1.4 ± 0.1, 1.3 ± 0.1, and 1.2 ± 0.1, respectively. The mean commissure height and Valsalva height were 18.6 ± 1.8 mm and 22.5 ± 2.4 mm, respectively. The mean gH was 14.9 ± 1.4 mm, with the longest gH of the non-coronary cusp. The mean eH was 8.5 ± 1.3 mm, with no significant difference among the 3 coronary cusps (*P* = .232) (**Figure 3**).

**Figure 3.**
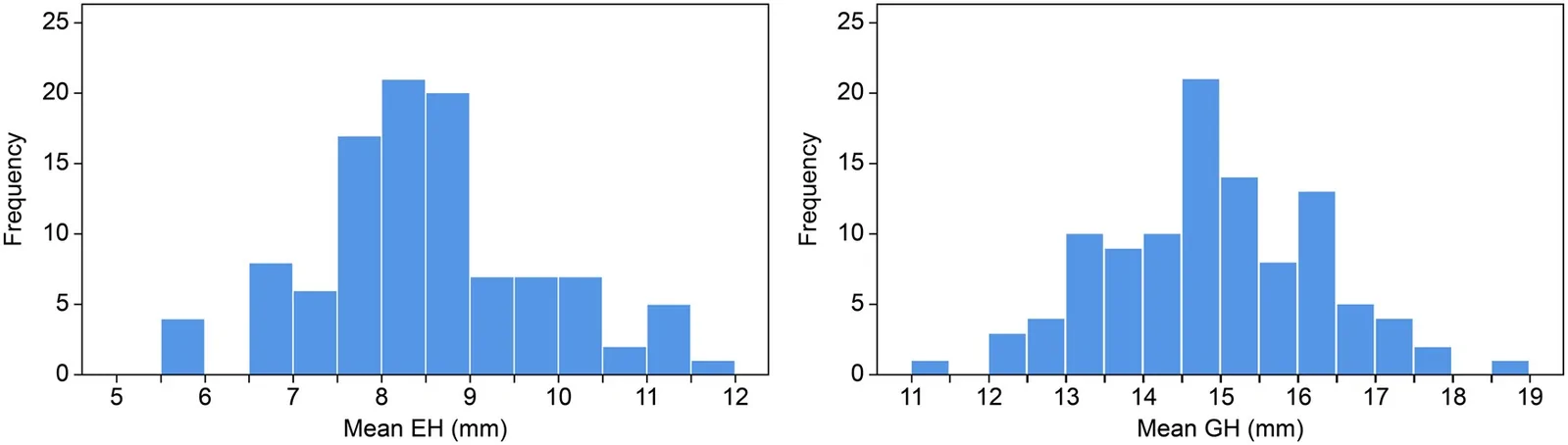
Histogram of the Mean Effective Height and Mean Geometric Height. Abbreviations: eH, effective height; gH, geometric height

**Table 2.** Measured Parameters

Measured parameter	*n* = 105	*P*-value (ANOVA)
Aortic annular diameter (mm)	24.2 ± 1.9	
SoV diameter (mm)		<.001
Right coronary SoV to the opposite interleaflet triangle	34.8 ± 3.1	
Left coronary SoV to the opposite interleaflet triangle	34.3 ± 3.0	
Non-coronary SoV to the opposite interleaflet triangle	35.4 ± 2.9	
Mean	34.8 ± 2.9	
Commissure diameter (mm)	32.3 ± 2.9	
Sinotubular junction diameter (mm)	30.1 ± 2.7	
Ascending aorta diameter (mm)	32.6 ± 3.0	
Commissure distance		<.001
Right CC	24.4 ± 2.3	
Left CC	26.1 ± 2.4	
Non-CC	24.3 ± 2.3	
Mean	24.9 ± 2.2	
Commissure height (mm)		<.001
Between right and left CCs	18.7 ± 2.0	
Between left and non-CCs	17.7 ± 1.9	
Between non- and right CCs	19.6 ± 2.3	
Mean	18.6 ± 1.8	
Valsalva height (mm)		.063
Right coronary SoV	23.0 ± 3.0	
Left coronary SoV	22.2 ± 2.5	
Non-coronary SoV	22.5 ± 3.3	
Mean	22.5 ± 2.4	
Geometric height (mm)		<.001
Right CC	14.4 ± 1.6	
Left CC	14.4 ± 1.5	
Non-CC	15.9 ± 1.8	
Mean	14.9 ± 1.4	
Effective height (mm)		.232
Right CC	8.5 ± 1.3	
Left CC	8.5 ± 1.3	
Non-CC	8.6 ± 1.4	
Mean	8.5 ± 1.3	

Abbreviations: ANOVA, analysis of variance; CC, coronary cusp; SoV, sinus of Valsalva.

The mean eH was weakly correlated with the mean body surface area (r = 0.332, *P* = .001) but not with the mean body height. A strong positive correlation was observed between the mean eH and gH (r = 0.728, *P* < .001) (**Figure 4**). Weak-to-moderate correlations were observed between the mean eH and mean sinus of Valsalva diameter, mean ST-J diameter, mean commissure height, and mean Valsalva height (r = 0.428, 0.552, 0.410, and 0.411, respectively) (all *P* < .001). However, there was no significant correlation between the mean eH and mean AN diameter (r = 0.119, *P* = .227).

**Figure 4.**
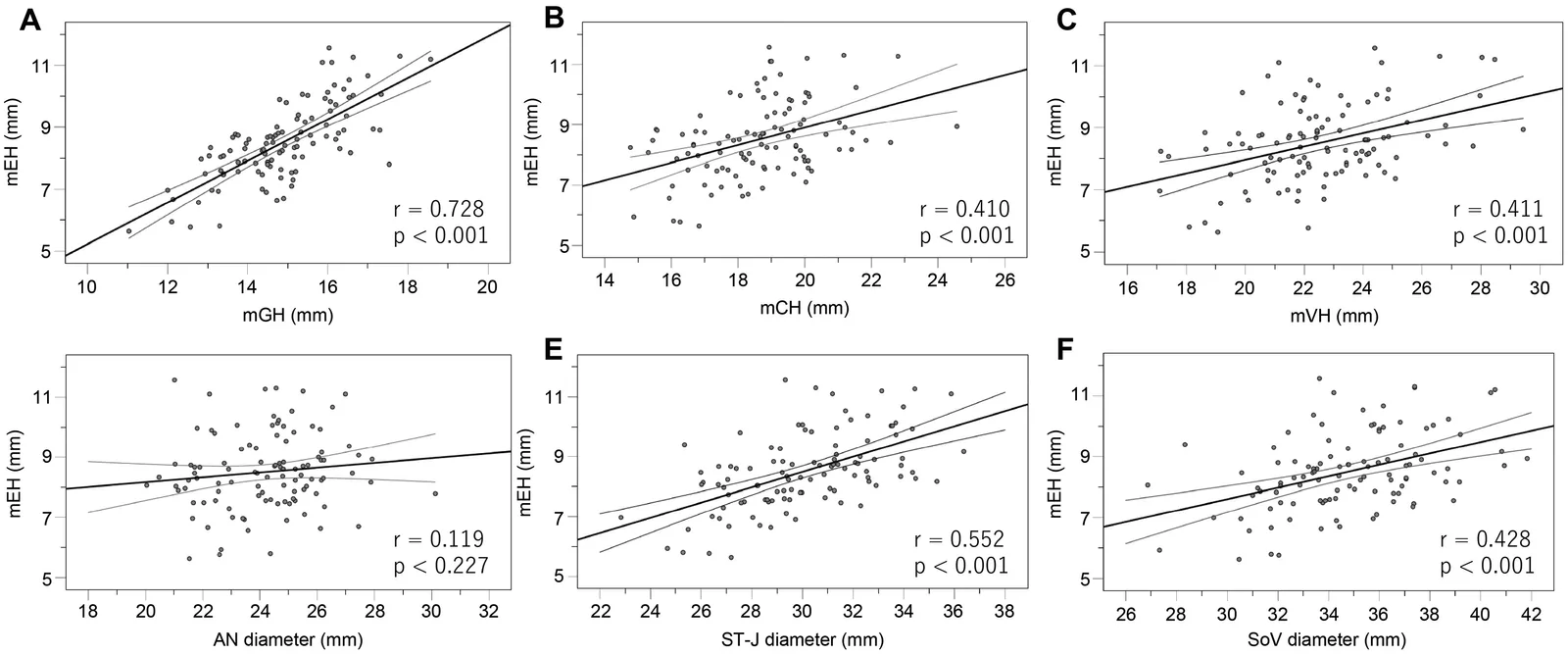
Association Between the eH and Other Parameters. The Solid Line Represents the Fitted Regression Line, and the Grey Lines Represent the Upper and Lower Limits of the 95% Confidence Interval. (A) meH and mgH. (B) meH and mCH. (C) meH and mVH. (D) meH and mean AN diameter. (E) meH and mean ST-J diameter. (F) meH and mean SoV diameter. Abbreviations: AN diameter, aortic annular diameter; eH, effective height; gH, geometric height; mCH, mean commissure height; meH, mean effective height; mgH, mean geometric height; mVH, mean Valsalva height; SoV, sinus of Valsalva; ST-J, sinotubular junction

In the multiple regression analysis, only the mean gH (*B* = 0.814; 95% confidence interval [CI]: 0.683 to 0.944; *P* < .001), mean AN diameter (*B* = −0.379; 95% CI, −0.466 to −0.292; *P* < .001), and ST-J diameter (*B* = 0.139; 95% CI, 0.077 to 0.200; *P* < .001) were retained in the final model and thus independently associated with eH (**Table 3**). Sex, mean body surface area, sinus of Valsalva diameter, commissure diameter, commissure height, and Valsalva height were excluded during the stepwise selection procedure. The final model had a coefficient of multiple determination (*R*^2^) of 0.739. Accordingly, the predicted eH (mm) can be expressed as follows: eH = 1.405 +0.814 × mean gH − 0.379 × mean AN diameter + 0.139 ×ST-J diameter. This equation represents the final regression model derived from the present cohort and facilitates the interpretation of the observed anatomical relationships. The variance inflation factor values for mean gH, mean AN diameter, and mean ST-J diameter were 1.969, 1.660, and 1.740, respectively, indicating no severe multicollinearity. **Table S1** presents a correlation matrix of the major CT-derived anatomical variables. **Figure S1** shows a scatterplot of the observed versus predicted eH values derived from the final multivariable regression model. The modelled effects of AN and ST-J diameters on predicted eH while holding the remaining variables constant are illustrated in **Figure S2A and B**, respectively.

**Table 3.** Multivariable Regression Analysis of Factors Associated With Effective Height

	Unstandardized coefficient		Standardized coefficient	*P*-value	95% CI		VIF
Variable	*B*	SE	*Β*		Lower	Upper	
(Constant)	1.405	0.894		.119	−0.368	3.179	
gH	0.814	0.066	0.881	<.001	0.683	0.944	1.969
AN diameter	−0.379	0.044	−0.567	<.001	−0.466	−0.292	1.660
ST-J diameter	0.139	0.031	0.300	<.001	0.077	0.200	1.740

*R* ^2^ = 0.739, ANOVA *P *< 0.001.

Abbreviations: AN, aortic annular; ANOVA, analysis of variance; CI, confidence interval; gH, geometric height; SE, standard error; ST-J, sinotubular junction; VIF, variance inflation factor.

The intra-class correlation coefficients for intra- and interobserver reliability of the mean eH, gH, AN diameter, and ST-J diameter were 0.979 and 0.908, 0.935 and 0.907, 0.98 and 0.976, and 0.993 and 0.939, respectively.

## DISCUSSION

We evaluated the characteristics of eH in the morphologically normal aortic valve complex during valve closure using ECG-gated cardiac CT. Our results demonstrated a strong positive association between eH and gH and showed that gH, AN diameter, and ST-J diameter were independently associated with eH.

Effective height was proposed as an objective parameter to assess the cusp configuration and can be measured with a calliper intraoperatively or by pre-, intra-, and postoperative echocardiography. The directly measured eH ranges from 8 to 10 mm in normal aortic valves,^3^ with a previous echocardiographic study demonstrating a normal eH of 9.3 ± 2.4 mm. Postoperative assessment recommended an eH of ≥9 mm for successful AVr, with the presumption that normalization of eH would lead to normal aortic valve function.^4^ A postoperative eH of <9 mm on echocardiography is a predictor of unfavourable long-term outcomes after valve-preserving aortic root repair.^6^ Thus, an eH of ≥9 mm is commonly used as a clinical important indicator for durable AVr.

We revealed that the mean eH of the normal tricuspid aortic valve was 8.5 ± 1.3 mm, similar to the findings of 2 previous CT studies (8.6-8.9 mm)^7^^,^^8^ and the anatomical investigation (8.5 mm).^9^ The mean eH of ≥9 mm, 8-9 mm, and <8 mm accounted for 28%, 39%, and 33% of our cases, respectively. Herein, the normal eH was not always an absolute value and less than the recommended value of 9 mm.

Reportedly, eH is strongly correlated with the body size of the surface area, weight and height, and the aortic root dimension of the aortoventricular, sinus of Valsalva, and ST-J diameter,^4^ highlighting the necessity of adjusting the targeted eH based on the individual.^10^ In the present analysis, eH demonstrated a strong positive correlation with gH, which showed considerable interindividual variability. gH represents the intrinsic amount of cusp tissue and serves as a quantitative indicator of cusp restriction or retraction.^11^ Accordingly, when the gH is small, attempting to achieve an excessively high eH may result in restricted cusp motion and inadequate central coaptation, potentially leading to residual regurgitation. This interpretation is consistent with the geometrical concept proposed by Schäfers et al, who indicated that the eH should be adjusted in proportion to the available cusp tissue to restore the physiological valve configuration.^4^^,^^12^ Taken together, our results suggest that eH should be interpreted in the context of individual leaflet geometry, particularly gH, and demonstrate that substantial interindividual variability exists in normal eH despite morphologically normal valve anatomy.

The root dimensions and cusp configuration were normalized to achieve normal aortic valve function. Although interference with root dimensions is presumed to affect valve configuration, particularly the cusp configuration, clinical evidence is less acknowledged. Herein, we also identified the anatomical components independently associated with eH and quantitatively characterized their relationships, thereby providing anatomical reference data for future investigations of AVr.

As expected, the gH showed the strongest positive association with eH. Among the root dimensions, the AN diameter was negatively associated with eH, whereas the ST-J diameter was positively associated. Although the AN diameter was not significantly associated with eH in the univariable analysis, its negative association became evident after adjustment for gH and ST-J diameter in the multivariable model. This finding likely reflects a suppression effect related to correlations among anatomically interrelated aortic root dimensions; however, the variance inflation factor values for gH, AN diameter, and ST-J diameter were all below 2.0, indicating no severe multicollinearity. These results are consistent with computer simulation findings, which indicated that the eH and coaptation height increased with increased gH and that the eH and coaptation height decreased with increased AN diameter.^5^^,^^13^ The influence of the ST-J diameter on the eH was not described in the other simulation study, which indicated that the coaptation height tended to decrease with an increased ratio of ST-J to aortic annulus diameter.^14^ However, the eH is assumed to increase with increased ST-J diameter, considering that the free edge of the cusp gets close to the straight line between the commissures, which leads to upward and outward displacement with the ST-J dilatation. Our findings suggest that eH is influenced by not only the native leaflet geometry but also the relationship between eH and aortic root dimensions, including the AN and ST-J diameters (**Figures S1, S2A,** and **S2B**). However, the regression model was derived from a single retrospective cohort using a stepwise variable selection procedure and should therefore be considered exploratory and hypothesis-generating. External validation in independent cohorts is required before these relationships can be translated into clinically applicable repair strategies or used to determine intraoperative repair targets.

An diameter >28 mm is a risk factor for late failure of valve-sparing aortic root replacement.^6^^,^^15^^,^^16^ Aortic annuloplasty has been added if the AN diameter is >26 mm.^17^ Similarly, aortic annuloplasty has been added to reduce the annulus diameter and increase the coaptation height if the annulus diameter is >25 mm, considering that a dilated annulus is a risk factor for intraoperative failure of the remodelling.^18^^,^^19^

Sinotubular junction dilatation also leads to aortic regurgitation. Reducing the ST-J diameter can restore aortic valve function.^20^^,^^21^ However, definite size criteria that interfere with the ST-J have not yet been established. Matsushima et al^11^ stated that a diameter of >35 mm may be too large for tricuspid aortic valves. Our study, in which the mean ratio of ST-J diameter to AN diameter was 1.2, might provide a reference for relative dimensional relationships, suggesting that an STJ diameter in this range could be considered in the context of annular dilatation, although further validation is required.

This study has some limitations, including its single-centre retrospective nature with a relatively small sample size, which may limit the generalizability of the findings. Second, the study cohort was predominantly male, further hindering generalizability to women. In addition, the participants comprised an older clinical cohort with a high prevalence of cardiovascular and vascular comorbidities, but with morphologically normal tricuspid aortic valves, and should not be considered a healthy reference population. Age-related valvular stiffening, reduced cusp mobility, and vascular remodelling associated with atherosclerotic changes may have influenced the observed geometric relationships of the aortic valve complex. The present findings may not be directly generalizable to younger patients undergoing elective AVr for isolated aortic regurgitation or aortic root aneurysms. Third, all measurements were obtained from ECG-gated cardiac CT images reconstructed at mid-diastole, which represents the static phase of the cardiac cycle. Therefore, the dynamic motion of the valve cusps and temporal variability of the eH could not be evaluated. Additionally, static CT-derived eH measurements may not fully reflect the functional reserve of cusp coaptation throughout the cardiac cycle. Future studies using cine-CT or 4 D-CT may provide further insights into the temporal behaviour and physiological relevance of eH. Fourth, although the eH was measured on standardized cross-sectional planes, potential errors related to image reconstruction and interobserver variability cannot be completely excluded. Fifth, cusp- and commissure-specific measurements were averaged to generate representative patient-level values for the main analyses. Although this approach was appropriate for evaluating patient-level determinants of eH, it may have obscured clinically relevant asymmetries among individual cusps and commissures, warranting further studies. Sixth, functional validation was not performed using echocardiography or intraoperative measurements of cusp coaptation, which offers an opportunity for future research. Seventh, because the regression model was derived using a stepwise procedure in a single retrospective cohort, it should be considered exploratory and requires external validation. Finally, this study did not include a comparative cohort with aortic valve, aortic root, or ascending aortic pathology. Future studies incorporating such comparative cohorts are warranted to determine the applicability of these findings in surgical decision-making.

Despite these limitations, the present study provides quantitative in vivo CT-based evidence regarding the anatomical determinants of eH in morphologically normal tricuspid aortic valves. Unlike previous conceptual and simulation-based investigations, our research demonstrates substantial interindividual variability in normal eH and quantitatively characterizes its relationships with gH and aortic root dimensions in a clinical cohort. Accordingly, the present findings should be considered anatomical reference data that may inform future research on individualized approaches to AVr.

## CONCLUSION

We demonstrated that eH was strongly associated with gH and independently associated with gH, AN diameter, and ST-J diameter in morphologically normal tricuspid aortic valves. These findings provide quantitative in vivo evidence regarding the anatomical determinants of eH and its variability among individuals. They may serve as anatomical reference data for future studies investigating individualized approaches to AVr, although further validation in dedicated repair cohorts is required.

## Supplementary Material

ivag233_Supplementary_Data

## Data Availability

The data underlying this article will be shared on reasonable request to the corresponding author.

## Abbreviations

AN diameter: aortic annular diameter
AVr: aortic valve repair
CI: confidence interval
CT: computed tomography
ECG: electrocardiography
eH: effective height
gH: geometric height
IRB: institutional review board
SE: standard error
ST-J: sinotubular junction
