# Supplementary material for: Determinants of Effective Height in Morphologically Normal Tricuspid Aortic Valves: A Computed Tomography-Based Anatomical Study
Source: Interdiscip Cardiovasc Thorac Surg. 2026 Aug 20;41(11):ivag233. doi: 10.1093/icvts/ivag233 (PMC13730563; doi:10.1093/icvts/ivag233)
Supplement: Supplementary file 1 [file ivag233_supplementary_data.zip › Supplementary Table S1.docx]

**Supplementary Table**

Table S1. Correlation matrix of major CT-derived anatomical variables

| Variable | eH | gH | CH | VH | AN diameter | SoV diameter | Commissure diameter | ST-J diameter |
| --- | --- | --- | --- | --- | --- | --- | --- | --- |
| eH | 1.000 | 0.728*** | 0.410*** | 0.411*** | 0.119 | 0.428*** | 0.529*** | 0.552*** |
| gH | 0.728*** | 1.000 | 0.638*** | 0.588*** | 0.600*** | 0.667*** | 0.651*** | 0.624*** |
| CH | 0.410*** | 0.638*** | 1.000 | 0.837*** | 0.604*** | 0.710*** | 0.624*** | 0.573*** |
| VH | 0.411*** | 0.588*** | 0.837*** | 1.000 | 0.528*** | 0.701*** | 0.721*** | 0.526*** |
| AN diameter | 0.119 | 0.600*** | 0.604*** | 0.528*** | 1.000 | 0.629*** | 0.556*** | 0.525*** |
| SoV diameter | 0.428*** | 0.667*** | 0.710*** | 0.701*** | 0.629*** | 1.000 | 0.858*** | 0.769*** |
| Commissure diameter | 0.529*** | 0.651*** | 0.624*** | 0.721*** | 0.556*** | 0.858*** | 1.000 | 0.881*** |
| ST-J diameter | 0.552*** | 0.624*** | 0.573*** | 0.526*** | 0.525*** | 0.769*** | 0.881*** | 1.000 |

Values are Pearson correlation coefficients (n = 105).

*p < 0.05; **p < 0.01; ***p < 0.001.

CT: computed tomography, eH: Effective height, gH: Geometric height, CH: Commissure height, VH: Valsalva height, AN: Aortic annular, SoV: Sinus of Valsalva, ST-J: Sinotubular junction.
