# Supplementary material for: Determinants of Effective Height in Morphologically Normal Tricuspid Aortic Valves: A Computed Tomography-Based Anatomical Study
Source: Interdiscip Cardiovasc Thorac Surg. 2026 Aug 20;41(11):ivag233. doi: 10.1093/icvts/ivag233 (PMC13730563; doi:10.1093/icvts/ivag233)
Supplement: Supplementary file 1 [file ivag233_supplementary_data.zip › Supplementary Figure S1 and S2 .docx]

Supplementary Figure S1

Supplementary Figure S2

 **Supplementary Figure Legends**
Supplementary Figure S1. Observed versus predicted effective height derived from the final multivariable regression model. The solid line represents the fitted regression line, and the grey lines represent the upper and lower limits of the 95% confidence interval.
EH: Effective height

Alt text: Scatterplot showing observed versus predicted effective height values derived from the final multivariable regression model. A fitted regression line and 95% confidence interval limits are displayed, demonstrating agreement between the observed and model-predicted values.

Supplementary Figure S2A. An illustration of the effect of aortic annular diameter on predicted effective height derived from the final multivariable regression model. Predicted eH values were calculated while geometric height and sinotubular junction diameter were fixed at their cohort mean values.

Alt text: Line plot illustrating the effect of aortic annular diameter on predicted effective height. Predicted effective height decreases as aortic annular diameter increases while geometric height and sinotubular junction diameter are held constant at their cohort mean values.

Supplementary Figure S2B. An illustration of the effect of sinotubular junction diameter on predicted effective height derived from the final multivariable regression model. Predicted eH values were calculated while geometric height and aortic annular diameter were fixed at their cohort mean values. EH: Effective height; ST-J: Sinotubular junction; AN diameter: Aortic annular diameter

Alt text: Line plot illustrating the effect of sinotubular junction diameter on predicted effective height. Predicted effective height increases as sinotubular junction diameter increases while geometric height and aortic annular diameter are held constant at their cohort mean values.
